# Rearrangement of Actin Cytoskeleton by Zika Virus Infection Facilitates Blood–Testis Barrier Hyperpermeability

**DOI:** 10.1007/s12250-020-00343-x

**Published:** 2021-02-03

**Authors:** Yiwen Nie, Lixia Hui, Moujian Guo, Wei Yang, Rui Huang, Junsen Chen, Xinyue Wen, Meng Zhao, Ying Wu

**Affiliations:** 1 State Key Laboratory of Virology, School of Basic Medical Sciences, Wuhan University, Wuhan, 430072 China; 2 Hubei Province Key Laboratory of Allergy and Immunology, Wuhan, 430071 China

**Keywords:** Zika virus (ZIKV), Envelope protein, Actin filaments, Sertoli cell barrier (SCB), Blood-testis barrier (BTB)

## Abstract

In recent years, various serious diseases caused by Zika virus (ZIKV) have made it impossible to be ignored. Confirmed existence of ZIKV in semen and sexually transmission of ZIKV suggested that it can break the blood–testis barrier (BTB), or Sertoli cell barrier (SCB). However, little is known about the underlying mechanism. In this study, interaction between actin, an important component of the SCB, and ZIKV envelope (E) protein domain III (EDIII) was inferred from co-immunoprecipitation (Co-IP) liquid chromatography–tandem mass spectrometry (LC–MS/MS) analysis. Confocal microscopy confirmed the role of actin filaments (F-actin) in ZIKV infection, during which part of the stress fibers, the bundles that constituted by paralleled actin filaments, were disrupted and presented in the cell periphery. Colocalization of E and reorganized actin filaments in the cell periphery of transfected Sertoli cells suggests a participation of ZIKV E protein in ZIKV-induced F-actin rearrangement. Perturbation of F-actin by cytochalasin D (CytoD) or Jasplakinolide (Jas) enhanced the infection of ZIKV. More importantly, the transepithelial electrical resistance (TEER) of an *in vitro* mouse SCB (mSCB) model declined with the progression of ZIKV infection or overexpression of E protein. Co-IP and confocal microscopy analyses revealed that the interaction between F-actin and tight junction protein ZO-1 was reduced after ZIKV infection or E protein overexpression, highlighting the role of E protein in ZIKV-induced disruption of the BTB. We conclude that the interaction between ZIKV E and F-actin leads to the reorganization of F-actin network, thereby compromising BTB integrity.

## Introduction

Zika virus (ZIKV), one of the mosquito-borne viruses, belongs to the genus *Flavivirus* of the family *Flaviviridae*. ZIKV infection causes a series of symptoms, ranging from mild illness (fever, joint pain) to severe clinical manifestations (multiple organ failure, meningitis). Distinct from other flaviviruses, ZIKV persists in multiple organs and causes unique symptoms, such as neonatal microcephaly in children, and Guillain-Barré syndrome, oligospermia, haematospermia in adults (Lessler *et al*. 2016; Musso and Gubler 2016; Pierson and Diamond 2018). ZIKV RNA can persist in semen and sperm for up to 370 days (Mlera and Bloom 2019) and can be transmitted sexually (D'Ortenzio *et al*. 2016). Both ZIKV MR766 and ZIKV Paraiba could establish persistent infection in human testicular cell lines, including Hs1.Tes and Sertoli cells (Mlera and Bloom 2019). Meanwhile, Kumar *et al*. (2018) reported that human Sertoli cells support high levels of ZIKV replication and persistence. Besides, studies using mouse models have revealed that testicular ZIKV infection has severe consequences, including testicular atrophy and spermatorrhea, which are closely related to male infertility (Govero *et al*. 2016; Ma *et al*. 2016; Sheng *et al*. 2017). Nevertheless, the mechanisms involved in blood–testis barrier (BTB) disruption and these phenomena remain to be identified.

The spherical ZIKV particles are 40–70 nm in diameter and encapsulate a positive-stranded RNA genome that encodes a single open reading frame. The viral polyprotein precursor is processed into three structural proteins (C, PrM/M, E) and seven non-structural proteins (NS1, NS2A, NS2B, NS3, NS4A, NS4B, NS5) by host and viral proteases. Notably, as the major antigenic determinant, E protein is also crucial for receptor binding and membrane fusion (Shi and Gao 2017). Further, several reports have highlighted the importance of E protein in virulence and pathogenesis. Fontes-Garfias *et al*. (2017) reported that glycosylation of ZIKV E protein is critical for ZIKV virulence in A129 mice and is required for ZIKV infection of the vector mosquito, *Aedes aegypti*. A recent study by Shan *et al.* (2020) revealed that an evolutionary mutation in the E protein (V473M) preceding the 2015 epidemic enhanced ZIKV virulence and fitness for transmission. Further, Zhou *et al*. (2019) showed that the interaction between ZIKV E protein and major facilitator superfamily domain-containing protein 2 (Mfsd2a) in human brain microvascular endothelial cells enhanced the ubiquitination and degradation of Mfsd2a, thus hindering docosahexaenoic acid intake mediated by Mfsd2a, and eventually resulting in growth restriction of the brain.

The actin skeleton, as an indispensable cellular component, is essential for maintaining an intact cell structure and for protein synthesis and cargo transport. Several studies have revealed that the actin skeleton is also crucial for infection of various viruses. For example, herpes simplex virus 1 causes biphasic changes in the actin filaments structure to facilitate target cell infection (Xiang *et al*. 2012). Dengue virus 2 (DENV2) induces the formation of filopodia that required for virus invasion in HMEC-1 cells (Zamudio-Meza *et al*. 2009). Vaccinia virus regulates actin nucleation to form a comet-like structure that facilitates virus spread between cells (Cudmore *et al.* 1997). Human immunodeficiency virus (HIV) triggers cortical actin dynamic changes to allow entry into resting CD4 T cells (Yin *et al.* 2020). Moreover, correlations between the cytoskeleton and flaviviruses, including West Nile virus (WNV), Japanese encephalitis virus (JEV), and DENV, have also been reported (Chu *et al.* 2003; Wang *et al.* 2010; Henry Sum 2015; Zhang *et al.* 2019). However, specific research on the role of actin cytoskeleton in terms of BTB disruption after ZIKV infection is lacking.

The testes, as the major male reproductive organ, are the site of spermatogenesis and androgens synthesis. The testis is divided into two separate compartments: a peritubular compartment, which is mainly comprised of Leydig cells and macrophages, and seminiferous tubules, which consist of germ cells at all stages of maturation, with the most mature cells lying closest to the central lumen, and supporting Sertoli cells (Mruk and Cheng 2015). The BTB or Sertoli cell barrier (SCB) is a compact structure mainly formed by tight and adherent junctions between Sertoli cells and are composed of transmembrane tight-junction (TJ) proteins such as ZO-1, occludin, and claudins, providing a microenvironment for normal sperm development (Cheng and Mruk 2012; Kaur *et al.* 2014). The most prominent feature of this structure is the large number of tightly packed actin filaments bundles between the Sertoli cells, which provide strong support for cell-to-cell junctions and render the BTB, one of the tightest blood-tissue barriers (Mok *et al.* 2012). Knockdown of Rictor (rapamycin-insensitive companion of mTOR), the key component of mTORC2 that is known to regulate actin cytoskeleton, was found to perturb Sertoli-cell TJ barrier function and BTB integrity by affecting F-actin organization (Mok *et al.* 2013). Furthermore, overexpression of integrin β3 and actin filaments rearrangement in endothelial cells induced by DENV2 contributed to the endothelial barrier dysfunction and vascular permeability observed in dengue hemorrhagic fever (Zhang *et al.* 2007). Based on these findings, we surmised that actin skeleton reorganization is likely to be involved in BTB disruption caused by ZIKV.

In this study, we sought to explore the role of the actin skeleton in ZIKV infection and BTB disruption, and whether viral protein is involved in these processes. Our data demonstrated that ZIKV infection induces actin filaments rearrangement and that ZIKV E protein is involved. Disruption of actin filaments dynamics benefits ZIKV infection. Upon either ZIKV infection or ZIKV E protein overexpression, the transepithelial electrical resistance (TEER) values, in an *in vitro* mouse SCB (mSCB) model were significantly reduced, and the interaction between actin and ZO-1 in Sertoli cells was weakened in a dose-dependent manner, which might account for the decline in TEER. Thus, ZIKV E plays an important role in the hyperpermeability of the *in vitro* mSCB model caused by ZIKV infection. Together, these findings provide insights into a possible mechanism of BTB destruction in ZIKV-infected mice.

## Materials and Methods

### Cells and Virus

The Sertoli cell line TM-4 was purchased from ATCC and cultured in Dulbecco’s modified Eagle medium (DMEM) (Gibco) supplemented with 10% (v/v) FBS (Gibco) and 1% (v/v) Penicillin Streptomycin (Gibco) at 37 °C in an atmosphere containing 5% CO_2_. The culture of human embryonic kidney cells (HEK293T), baby hamster syrian kidney cells (BHK21) and Vero cells is consistent with Sertoli cells. Zika virus (strain *ZIKA-SMGC-1*, GenBank accession number: KX266255) was kindly provided by Dr. George F. Gao (Institute of Microbiology, Chinese Academy of Sciences) and propagated in Vero cells.

### Antibodies, Chemical Reagents and Plasmids

The primary antibodies used included a rabbit polyclonal antibody (pAb) against the ZIKV E protein (133314, Genetex), an anti-actin (Cell Signaling Technology) rabbit monoclonal antibody (mAb), a monoclonal mouse anti-glyceraldehyde 3-phosphate dehydrogenase antibody (GAPDH, 60004-1-Ig, proteintech), an anti-Flag antibody (F3165, Sigma-Aldrich), an anti-HA antibody (H9658, Sigma-Aldrich), an anti-mouse IgG antibody (Sigma-Aldrich), and an anti-ZO-1 rabbit pAb (21773-1-AP, proteintech).

The secondary antibodies used included a horseradish peroxidase (HRP)-conjugated anti-mouse IgG antibody and a HRP-conjugated anti-rabbit IgG antibody, and were all purchased from Jackson ImmunoResearch. Tetramethylrhodamine B isothiocyanate (TRITC)-phalloidin (Invitrogen) was used to label F-actin. FITC-ZIKV-E (FITC-Z6) antibody was kindly provided by Dr. George F. Gao and used to label Zika viruses. DyLight™ 488 donkey anti-rabbit IgG antibody (406416, Biolegend), Alexa Fluor 488 goat anti-mouse IgG antibody (405319, Biolegend). 4′, 6-diamidino-2-phenylindole (DAPI, P0131, Beyotime) was used to dye the nuclei. The actin skeleton inhibitor cytochalasin D (CytoD) (PHZ1063) and Lipofectamine™ 3000 transfection reagent were purchased from Invitrogen Corporation. Jas (ab141409) was purchased from abcam.

ZIKV-E(1–133), ZIKV-E(134–301) and ZIKV-EDIII genes were cloned into pcDNA3.1(+)-Flag vector, respectively. Primers were designed as follows: pcDNA3.1(+)-Flag-ZIKV-E(1–133)-F 5′-CGGGGTACCATCAGGTGCATCGGCGTGTCCAACAG-3′, pcDNA3.1(+)-Flag-ZIKV-E(1–133)-R 5′-CCGCTCGAGTTACTCAGGCTGGATGCTTTTGCCGG-3′; pcDNA3.1(+)-Flag-ZIKV-E(134–301)-F 5′-CGGGGTACCAACCTGGAATACAGGATCATGCTCAG-3′, pcDNA3.1(+)-Flag-ZIKV-E(134–301)-R 5′-CCGCTCGAGTTACTTCAGCCTCAGCTTGTCCATCT-3′; pcDNA3.1(+)-Flag-ZIKV-EDIII(302–405)-F 5′-CGGGGTACCGGCGTGAGCTATTCCCTGTGCACCGC-3′, pcDNA3.1(+)-Flag-ZIKV-EDIII(302–405)-R 5′- CCGCTCGAGTCAGCTGCCGCTCCTATGCCAATGGT -3'.

### Transfection

Transfection can be performed when the cells grow to 70%–80% confluence. The plasmid and transfection reagent were mixed in opti-MEM (Gibco) after incubation for 20 min, added dropwise to the cells, gently shaken and mixed. For transfection of HEK293T cells, Transporter^TM5^ transfection reagent (Polysciences) is sufficient, and the ratio of plasmid to transfection reagent is 1:2; for Sertoli cell line TM-4 and primary mouse Sertoli cells (mSCs), transfection is performed with Lipofectamine™ 3000 transfection reagent (Invitrogen) and the ratio of plasmid to transfection reagent is 1:2.5.

### Western Blotting

For Western blotting (WB), the total amount of sample protein is determined by Pierce BCA Protein Assay Kit (Thermo). The samples were subjected to sodium dodecyl sulfate-polyacrylamide gel electrophoresis (SDS-PAGE) and transferred to 0.45 μm pore size polyvinylidene difluoride (PVDF) membranes (Millipore) as described previously (Hui *et al.* 2020).

### Protein Expression

ZIKV EDIII was fused to the Fc region of human IgG, and soluble version of EDIII-Fc protein was expressed and purified. In briefly, the psectag-2A-EDIII-Fc plasmid was transfected with HEK293T cells, and replaced with serum-free DMEM after 7 h of transfection. After 48 h of expression, the supernatant was collected and purified with protein A-sepharose beads (GE, 29024680AD). Equilibrating the column with 20 mmol/L Na_3_PO_4_ (pH 7.2), the sample was subsequently applied and eluted with 0.1 mol/L glycine (pH 3.0). After testing the concentration and purity of the concentrated protein, perform subsequent experiments.

### Immunoprecipitation and Protein Identification

For immunoprecipitation, plates of Sertoli cells were washed twice with phosphate-buffer saline (PBS) and the cells were resuspended in pre-cooled IP-lysis buffer (20 mmol/L Tris–HCl pH 7.5, 150 mmol/L NaCl, 20 mmol/L KCl, 1.5 mmol/L MgCl_2_, 1% Triton X-100, 10% glycerol, 0.5 mol/L PMSF). The cells were lysed at 4 °C on rotary mixer for 1 h, followed by centrifugation at 13,500 ×*g* for 10 min. Take the supernatant and mix with 100 μL protein A-sepharose beads to remove non-specific binding. Subsequently centrifugation and supernatant was divided into two equal parts. One part was added with the CD80-Fc protein as a negative control, and the other part was added with the ZIKV-EDIII-Fc protein. Incubated at 4 °C overnight and then mixed with 200 μL protein A-sepharose beads for another 2 h. The pellets were washed three times with IP-lysis buffer and resuspended in 2 × SDS sample loading buffer and bathed in boiling water for 7 min. The samples were applied to SDS-PAGE under reducing conditions. Gels were subsequently stained by coomassie brilliant blue. Bands of interest were excised and subjected to LC–MS/MS and proteins identified by search of the UniProt database.

To confirm the interaction between actin and EDIII, briefly, pcDNA3.1(+)-Flag-ZIKV-EDIII and pcaggs-HA-M-actin plasmid were co-transfected into HEK293T cells. HEK293T cells were lysed in lysis buffer composed of 50 mmol/L Tris–HCl [pH 7.2–7.4], 5 mmol/L EDTA, 300 mmol/L NaCl, 10% Glycerol, protease inhibitor (Roche) for 1 h on rotary mixer at 4 °C, followed by centrifugation at 13,500 ×*g* for 10 min. Take the supernatant and incubate with the primary antibody overnight at 4 °C, normal mouse IgG antibody as a negative control. Subsequently, 60 μL of protein A-sepharose beads was added and incubated for 2 h at 4 °C. Then collected the beads and washed three times with lysis buffer. The samples were subjected to SDS-PAGE and proceeded WB.

### Confocal Immunofluorescence Microscopy

For laser scanning confocal immunofluorescence microscopy, the cells were fixed in 4% paraformaldehyde (Biosharp) for 15 min and permeated by 0.2% Triton X-100 (PBS) for 5 min. After washing three times with PBS and blocking with 3% bovine serum albumin (PBS) at room temperature for 1 h, the cells were stained with TRITC-phallodin (1:1000) and FITC-Z6 (1:1000) in 1% BSA-PBS for 1 h at room temperature. Then the dyes were removed and the cells were washed three times in PBS, and finally the nuclei were stained with DAPI. Fluorescence images were captured by laser scanning confocal immunofluorescence microscope (Leica SP8) under 63 × oil immersion objective. Images were processed using Adobe Photoshop (Adobe, CA).

### Quantitative RT-PCR

Total RNA was extracted with TRIzol reagent (Invitrogen) and the concentration was detected by NanoDrop (Thermo scientific). Quantitative RT-PCR (qRT-PCR) was performed with iTaq Universal SYBR Green Supermix (Bio-Rad Laboratories) on a Bio-Rad CFX96 Real-Time PCR System after reverse transcription using Moloney murine leukemia virus reverse transcriptase (Promega). Serially diluted expression plasmids containing the coding sequence of ZIKV-E were used to establish standard curve. The primers used to amplify corresponding genes were designed as follows: ZIKV-F 5′-TGAYAAGCARTCAGACAC-3′ and ZIKV-R 5′-TCACCARRCTCCCTTTGC-3′; mouse GAPDH-F 5′-TGTGTCCGTCGTGGATCTGA-3′ and mouse GAPDH-R 5′-TTGCTGTTGAAGTCGCAGGAG-3′; mouse actin-F 5′-GGCTGTATTCCCCTCCATCG-3′ and mouse actin-R 5′-CCAGTTGGTAACAATGCCATGT-3'.

### Cytotoxicity Test

To examine the cytotoxicity of drugs, Sertoli cells were seeded into 96-well plates (Thermo), cultured overnight to form a monolayer, corresponding concentrations of CytoD or Jas were added to cells and incubated for 48 h. The cell viability was determined by CCK8 assay (Cell Counting Kit-8, Biosharp) and calculated by the optical density (OD) values which were measured at a wavelength of 450 nm on a microplate reader (Thermo), at least 4 replicates per treatment and the experiment was repeated three times. The cell viability was expressed as the relative OD_450nm_, which represents the ratio of optical density (OD) as follows: (OD_450nm_ experimental condition − OD_450nm_ medium alone)/(OD_450nm_ untreated SCs − OD_450nm_ medium alone).

### Viral Plaque Assay

To determine the effects of the drugs on viral post entry step, Sertoli cells were seeded into 6-well tissue culture plates and cultured overnight in medium. The cells were infected with ZIKV (MOI = 1) for 2 h, and after washing the unabsorbed viruses with PBS, CytoD or Jas at the indicated concentrations were added. At 30 h post infection (hpi), the supernatants were collected and virus titers were determined by a plaque assay on BHK21 cell monolayers, as previously reported (Nikolay *et al.* 2018).

### Isolation of Primary mSCs and TEER Assay

Isolation of primary mSCs has been described previously (Hui *et al.* 2020). For TEER assay, primary mSCs were cultured in 6 cm dishes with 10% FBS DMEM/F-12 medium (Gibco) until monolayer was formed, then seeded cells on 12-well transwell polycarbonate membrane system (3402, Corning Life Sciences) for experiment. The cells were digested with 0.125% trypsin (Gibco) and evenly seeded at a density of 2.5 × 10^5^ per well. The TEER was measured daily by using epithelial volt/ohm meter (Evomx) with “chopstick” electrodes (World Precision Instrument). Experiments were performed when TEER reaches 50 Ω · cm^2^ or more and remains unchanged, which indicates 100% cell confluency. At least 3 replicate wells were designed for each set and 3 independent experiments were repeated. The SCB permeability was expressed as the relative TEER, which represents the ratio of resistance values (Ω) as follows: (Ωexperimental condition − Ωmedium alone)/(Ωmock − Ωmedium alone).

### Statistical Analysis

Data are presented as mean ± standard deviations (SD) and analyzed using GraphPad Prism software (GraphPad Software Inc., La Jolla, CA). The one-way ANOVA or two-way ANOVA was used for statistical analysis to compare the differences among treatment groups. *P* < 0.05 was considered statistically significant. All experiments were repeated at least three times.

## Results

### ZIKV EDIII-interacting Proteins

**Fig. 1:**
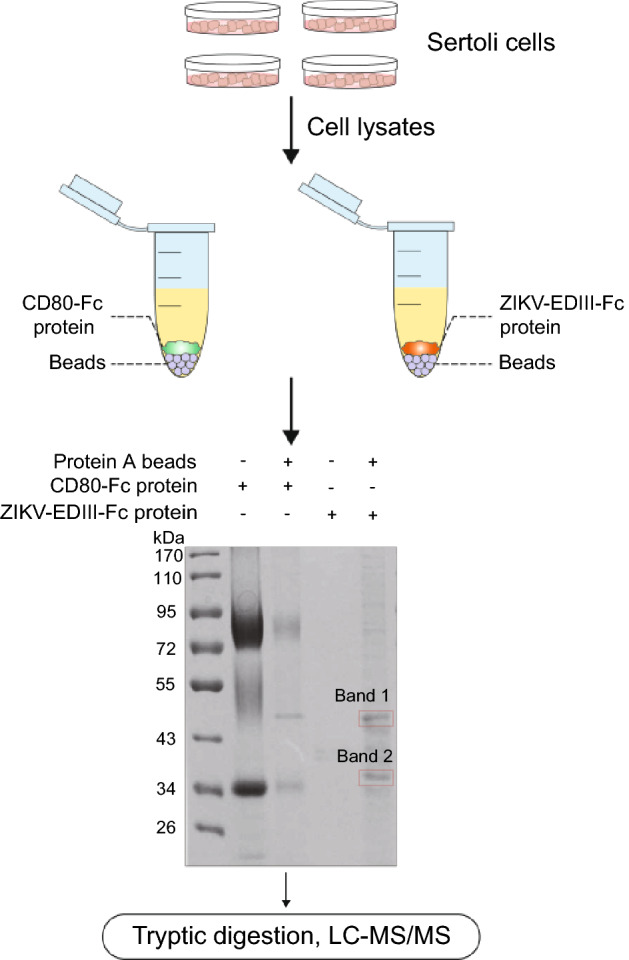
Flow chart of identification of ZIKV-EDIII protein interacting proteins. Sertoli cells lysates were incubated with CD80-Fc and protein A-sepharose beads or EDIII-Fc and protein A-sepharose beads, respectively. Samples of CD80-Fc and ZIKV-EDIII-Fc affinity-isolated proteins from Sertoli cell lysates were subjected to gel and performed coomassie blue staining. Band 1 and Band 2 excised from the gel and the whole affinity-isolated proteins eluted from protein A-sepharose beads were all subjected to LC–MS/MS analysis. By performing a sequence alignment with the Uniprot database, we obtained a number of potential interacting proteins, including actin and actin related proteins (Table 1).

To discover cellular interaction partners of ZIKV E protein, we affinity-isolated proteins from mouse Sertoli cells using a ZIKV EDIII-Fc chimeric protein and we examined these proteins by SDS-PAGE. As shown in Fig. 1, bands of interest in the SDS gel and the overall affinity-isolated proteins eluted from protein A beads were subjected to LC–MS/MS analysis. We thus identified a number of potential interaction partners, including actin and actin-related proteins (Table 1).

**Table 1:** LC–MS/MS identified interacting partners of ZIKV-EDIII protein.

Name	Mass(kDa)	%Cov(95)	Accession
*ZIKV-EDIII-Fc sepharose beads proteins*			
Actin, cytoplasmic 1	41.737	15.73	P60710
Myosin light polypeptide 6	16.930	9.18	Q60605
*Band 1*			
Tubulin beta-5 chain	49.671	22.75	P99024
Beta-actin-like protein 2	42.004	23.94	Q8BFZ3
*Band 2*			
Tropomyosin alpha-1 chain	32.681	70.42	P58771
Actin, cytoplasmic 1	41.737	30.13	P60710
Capping protein (Actin filament) muscle Z-line, alpha 1	32.954	5.59	Q5RKN9

### ZIKV E Interacts with Actin

**Fig. 2:**
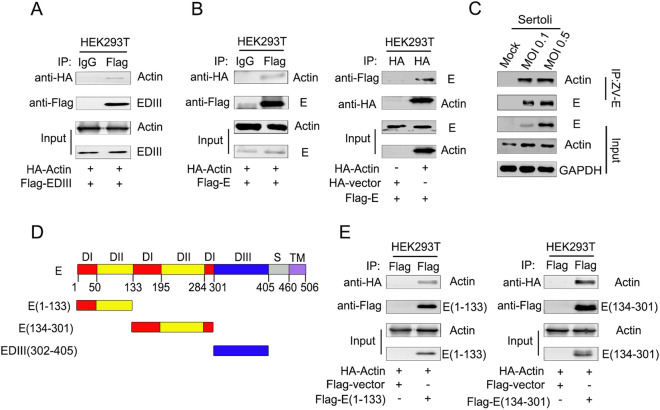
ZIKV E interacts with actin. **A** Co-IP of ZIKV EDIII protein and actin *in vitro*. Plasmid pcDNA3.1(+)-Flag-EDIII and pcaggs-HA-actin were co-transfected into HEK293T cells, the samples were collected at 48 h post transfection. Cell lysates were immunoprecipitated with anti-mouse immunoglobulin G (IgG) and anti-Flag antibodies, followed by SDS-PAGE and immunoblotting with anti-HA antibody. **B** Co-IP of ZIKV E protein and actin *in vitro*. Plasmid pcDNA3.1(+)-Flag-E and pcaggs-HA-actin were co-transfected into HEK293T cells, the samples were collected at 48 h post transfection. Cell lysates were immunoprecipitated with anti-mouse immunoglobulin G (IgG) and anti-Flag antibodies, followed by SDS-PAGE and immunoblotting with anti-HA antibody. HEK293T cells were co-transfected with empty vector, pcDNA3.1(+)-Flag-E and pcaggs-HA-actin, cells lysates were immunoprecipitated with anti-HA antibody followed by SDS-PAGE and immunoblotting with anti-Flag antibody. **C** Co-IP of ZIKV E protein and actin after viral infection. Sertoli cells were un-infected (mock) or infected with ZIKV at an MOI of 0.1 and 0.5, the samples were collected at 48 hpi. Cell lysate was immunoprecipitated with anti-ZIKV E antibodies followed by SDS-PAGE and immunoblotting with anti-actin antibody. **D** Diagrammatic representation of ZIKV E protein and three truncated constructs generated in this study. **E** Co-IP analysis of ZIKV E protein's actin interaction region. HEK293T cells were cotransfected with pcDNA3.1(+)-Flag-E(1–133) and pcaggs-HA-actin or pcDNA3.1(+)-Flag-E(134–301) and pcaggs-HA-actin. Cells lysates were immunoprecipitated with anti-Flag antibodies followed by SDS-PAGE and immunoblotting with anti-HA antibody.

It has been reported that ZIKV EDIII is the putative region responsible for receptor binding and has an important role in membrane fusion in most flaviviruses (Shi and Gao 2017). In this study, LC-MS/MS identified a number of potential interaction partners of ZIKV EDIII, including actin. The interaction between ZIKV EDIII and actin was verified by Co-IP analysis (Fig. 2A). Meanwhile, Co-IP and reverse Co-IP identified that ZIKV E interacted with actin (Fig. 2B). The amount of E proteins associated actin increased with increasing multiplicity of infection (Fig. 2C), which was consistent with the findings in cells cotransfected with E and actin expression plasmids as shown in Fig. 2B. To determine whether EDIII is the only region participating in the interaction with actin, E truncated constructs were generated (Fig. 2D) and assessed by Co-IP using lysates of transfected Sertoli cells. Surprisingly, ZIKV E regions containing residues 1–133 and 134–301, were both found to bind with actin, suggesting that not only EDIII, but also sequences present in E protein domain I (EDI) and/or E protein domain II (EDII) interact with actin (Fig. 2E).

### ZIKV Infection Induces Actin Filaments Rearrangement in Sertoli Cells

**Fig. 3:**
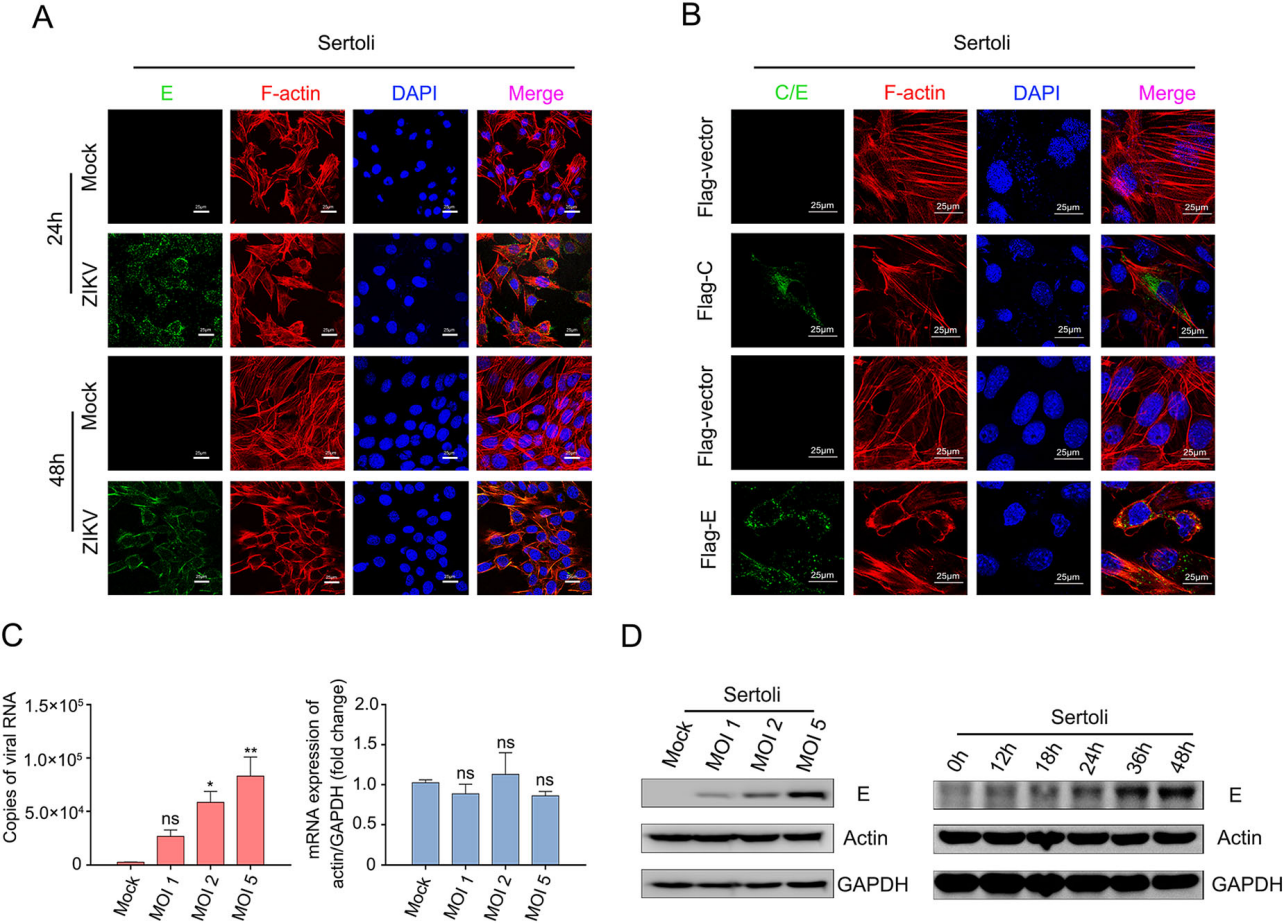
ZIKV infection induces actin filaments rearrangement in Sertoli cells. **A** Confocal microscopy analysis of Sertoli cells actin filaments skeleton under continuous ZIKV infection. Sertoli cells were un-infected (mock) or infected with ZIKV (MOI = 1). The cells were fixed in 4% paraformaldehyde at indicated time points, permeabilized, and stained with DAPI to label nuclei (blue), TRITC-phalloidin to label F-actin (red), and ZIKV (green) were detected by FITC-Z6 antibodies. The results are representative of three separate experiments. The scale bar indicates 25 μm. **B** Confocal microscopy analysis of Sertoli cells actin filaments skeleton after transfected with pcDNA3.1(+)-Flag-E plasmid. Sertoli cells were transfected with 2 μg empty vector, 2 μg pcDNA3.1(+)-Flag-C plasmid or 2 μg empty vector, 2 μg pcDNA3.1(+)-Flag-E plasmid, and fixed at 48 h post transfection. Then cells were stained with DAPI to label nuclei (blue), TRITC-phalloidin to label F-actin (red), Flag antibodies (green) to label capside protein and ZIKV-E (green) were detected by FITC-Z6 antibodies. The results are representative of three separate experiments. The scale bar indicates 25 μm. **C** Measurements of actin mRNA levels after ZIKV infection. Sertoli cells were un-infected (Mock) or infected with ZIKV at an MOI of 1, 2 and 5, and samples were collected at 30 hpi. Total RNA were extracted, copies of viral RNA and actin were measured by qRT-PCR. The results are representative of three separate experiments. Each value represents the mean ± SD of 3 separate replicates. *, *P* < 0.05; **, *P* < 0.01; ns, not significant (one-way ANOVA). **D** Measurements of actin protein levels after ZIKV infection. Sertoli cells were infected with ZIKV (MOI = 1) and collected at indicated time points, actin protein levels were examined by WB. 0 h: un-infected cells.

Several studies have shown that WNV, JEV, and DENV2 utilize the actin filament network in mammalian cells in the course of infection (Lee and Ng 2004; Henry Sum 2015; Cuartas-Lopez *et al.* 2018), but direct functional evidence of a role of actin during BTB disruption caused by ZIKV infection is lacking. Thus, Sertoli cells were infected with ZIKV at an MOI of 1, and the actin filament structures were monitored at various time points by confocal microscopy. As shown in Fig. 3A, after 24 h of infection, the organized stress fibers in Sertoli cells were partially disrupted when compared with those in mock control cells, and colocalization of E and F-actin was observed. With the progression of infection, the F-actin configuration in Sertoli cells became disorganized at 48 hpi, actin filaments were no longer distributed evenly across the cytoplasm and most stress fibers were disrupted. These results showed that ZIKV infection disrupts the integrity of the actin filament structure, which might benefit infection.

Next, we investigated whether ZIKV E would be involved in the reorganization of actin filaments caused by ZIKV infection. Confocal immunofluorescence microscopy revealed that transient expression of E proteins in transfected Sertoli cells resulted in the disruption of actin filament structure and the expressed proteins colocalized with actin when compared with empty vector or ZIKV capsid plasmid transfected cells (Fig. 3B). This finding was consistent with the viral infection results, indicating that E protein plays a crucial role in ZIKV infection in addition to host-cell receptor binding and membrane fusion. Besides, ZIKV infection did not alter the mRNA and protein levels of actin, as shown in Fig. 3C, D, indicating that ZIKV infection only induces structural disorganization of F-actin, without altering its expression.

### Disruption of Actin Filaments Dynamics Benefits ZIKV Infection

**Fig. 4:**
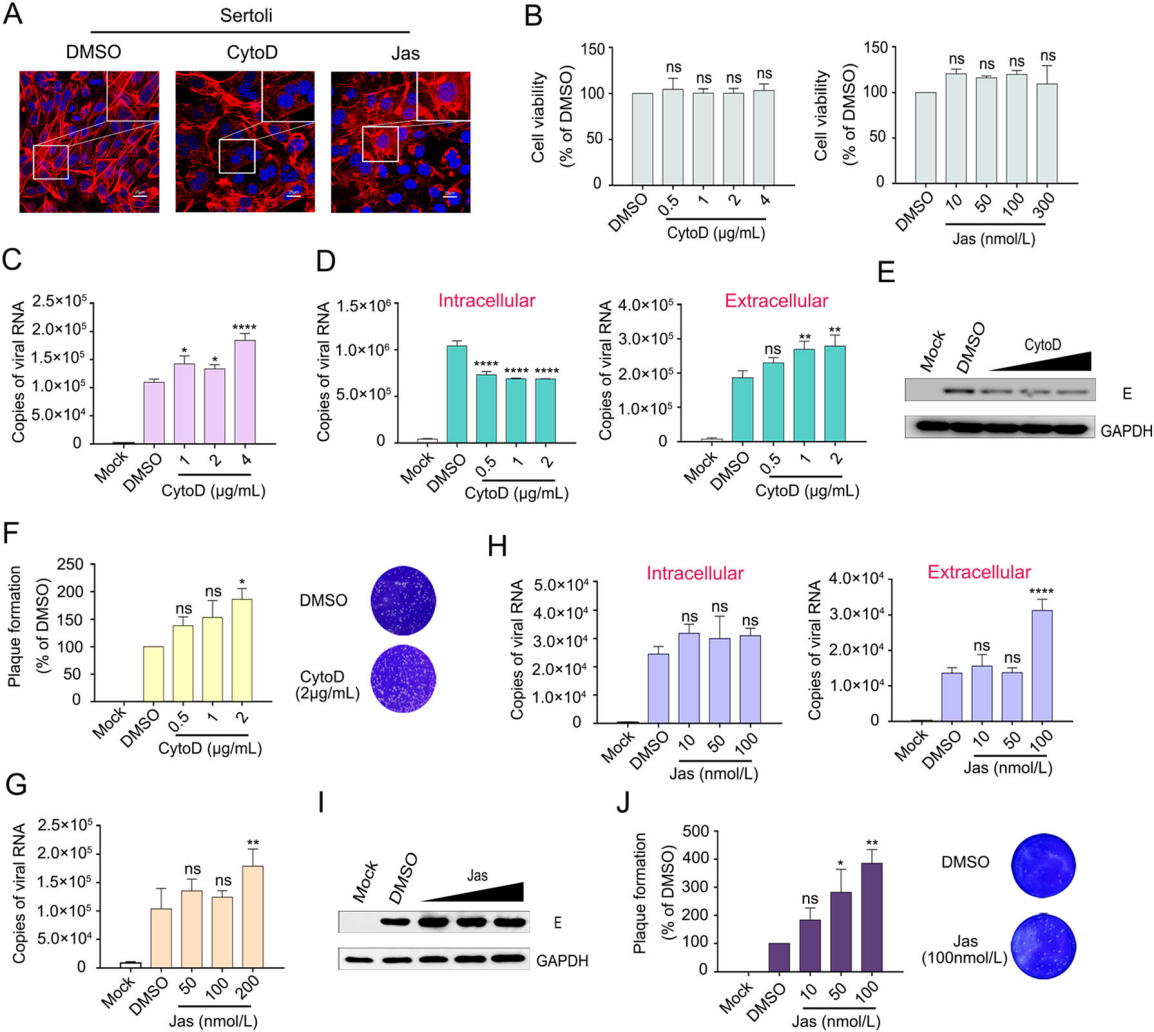
ZIKV infection was promoted by CytoD or Jas treatment. **A** Effects of CytoD and Jas on F-actin. Sertoli cells were treated with 2 μg/mL of CytoD or 200 nmol/L Jas for 26 h. Then cells were stained with DAPI to label nuclei (blue), TRITC-phalloidin to label F-actin (red). Control: DMSO treated cells. The scale bar indicates 25 μm. **B** Cytotoxicities of CytoD and Jas on Sertoli cells. Sertoli cells were incubated with CytoD or Jas at different concentrations for 48 h, and the cell viability was determined by CCK8 assay. Control: DMSO treated cells. The cell viabilities were expressed as the relative values to control, the results are representative of three separate experiments. Each value represents the mean ± SD of 4 separate replicates. ns, not significant (one-way ANOVA). **C** Effects of CytoD on ZIKV invasion of Sertoli cells. Sertoli cells were pretreated with DMEM containing 0.4% DMSO (control) or CytoD at 1, 2, 4 μg/mL for 3 h and after that infected with ZIKV (MOI = 1) in the absence of drugs for 2 h, discard the supernatants and wash three times with PBS, the cell samples were collected with TRIzol and measured by qRT-PCR. The results are representative of three separate experiments. Each value represents the mean ± SD of 3 separate replicates. *, *P* < 0.05; ****, *P* < 0.0001 (one-way ANOVA). **D–F** Effects of CytoD on ZIKV post entry step. Sertoli cells were incubated with ZIKV (MOI = 1) for 2 h and the supernatants were discarded, washed three times with PBS, then incubated with CytoD at the corresponding concentration. The cell and supernatant samples were collected at 30 hpi. Measured by qRT-PCR, WB and plaque assay. The results are representative of three separate experiments. Each value represents the mean ± SD of 3 separate replicates. *, *P* < 0.05; **, *P* < 0.01; ****, *P* < 0.0001; ns, not significant (one-way ANOVA). **G** Effects of Jas on ZIKV invasion of Sertoli cells. Sertoli cells were pretreated with DMEM containing 0.2% DMSO (control) or Jas at 50, 100, 200 nmol/L for 3 h and after that infected with ZIKV (MOI = 1) in the absence of drugs for 2 h, discard the supernatants and wash three times with PBS, the cell samples were collected with TRIzol and measured by qRT-PCR. The results are representative of three separate experiments. Each value represents the mean ± SD of 3 separate replicates. **, *P* < 0.01; ns, not significant (one-way ANOVA). **H-J** Effects of Jas on ZIKV post entry step. Sertoli cells were incubated with ZIKV (MOI = 1) for 2 h and the supernatants were discarded, washed three times with PBS, then incubated with Jas at the corresponding concentration. The cell and supernatant samples were collected at 30 hpi. Measured by qRT-PCR, WB and plaque assay. The results are representative of three separate experiments. Each value represents the mean ± SD of 3 separate replicates. *, *P* < 0.05; **, *P* < 0.01; ****, *P* < 0.0001; ns, not significant (one-way ANOVA).

To investigate the role of the actin cytoskeleton in ZIKV infection further, Sertoli cells were treated with CytoD, which acts as an actin polymerization inhibitor and disrupts the existing actin cytoskeleton, or Jas, which is a potent inducer of actin polymerization and depletes the cellular pool of free actin monomers available for *de novo* polymerization (Xiang *et al.* 2012). A previous study showed that CytoD treatment severely disrupts actin network, increases the number of actin filament ends, and leads to the formation of punctate actin foci in BSC-1 African green monkey kidney cells (Schliwa 1982). We observed similar effects in CytoD-treated Sertoli cells by confocal microscopy, CytoD disrupted the stress fibers and the actin cell cortex as compared to the DMSO control. In Jas-treated Sertoli cells, actin filaments were interrupted and piled up disorderly at the edge of the cell (Fig. 4A), demonstrating the feasibilities of the drugs. CCK8 assays revealed that the Sertoli cell viability was 104%, 100%, 100% and 103% after 48-h incubation with 0.5, 1, 2 and 4 μg/mL CytoD, respectively; and the ratio was 120%, 115%, 120%, and 109% when incubated with 10, 50, 100, and 300 nmol/L Jas, respectively, which were not significantly different from the control (Fig. 4B), indicating that the concentrations of CytoD or Jas used in this study had no effect on cell viability.

Sertoli cells were pretreated with CytoD and Jas at different concentrations for 3 h and infected with ZIKV (MOI = 1) for another 2 h in the absence of CytoD or Jas. Then, the cells were washed with PBS three times to remove the uninfected ZIKV and cell samples were collected with TRIzol. As shown in Fig. 4C, G, the amount of invading viruses increased in a dose-dependent manner; viral mRNA levels were 1.3-, 1.22-, and 1.69-fold increased after pretreatment with 1, 2, and 4 μg/mL of CytoD (Fig. 4C), the viral mRNA levels were also increased with increasing Jas concentration (Fig. 4G). These observations were striking, considering that the entry of other flaviviruses into cells was inhibited after CytoD or Jas treatment of the cells (Zhang *et al.* 2019). Therefore, we further investigated the impact of an interrupted actin cytoskeleton on viral post entry step. Sertoli cells were incubated with ZIKV for 2 h to allow the viruses to fully enter the cells. Then, the viruses were removed by washing and the cells were incubated with CytoD or Jas at the indicated concentrations. Control cells were treated with DMSO. Cell and supernatant samples were collected at 30 hpi. Virus contents in the cells were detected by qRT-PCR and WB, and the supernatants were assessed by qRT-PCR and viral plaque assay. As shown in Fig. 4D, E, the intracellular virus content decreased with increasing drug concentration. In detail, viral mRNA level was decreased by 0.6-fold in cells treated with 2 μg/mL CytoD. Instead, the virus content in the supernatant increased, and this was consistent with the plaque assay results (Fig. 4F). Although there was no difference in intracellular virus content between DMSO and Jas, the extracellular virus content increased significantly after Jas treatment (Fig. 4H–4J). These findings suggested that disturbance of the F-actin network enhanced the production of extracellular virus particles.

The ability of CytoD to enhance ZIKV infection was consistently detected over a range of virus dilutions, ensuring that this enhancement was not a result of the usage of different MOIs (data not shown). Based on the collective findings, we surmised that the disruption of actin filaments dynamics benefits ZIKV infection.

### ZIKV E Participates in the Hyperpermeability of an *In Vitro* mSCB Model

**Fig. 5:**
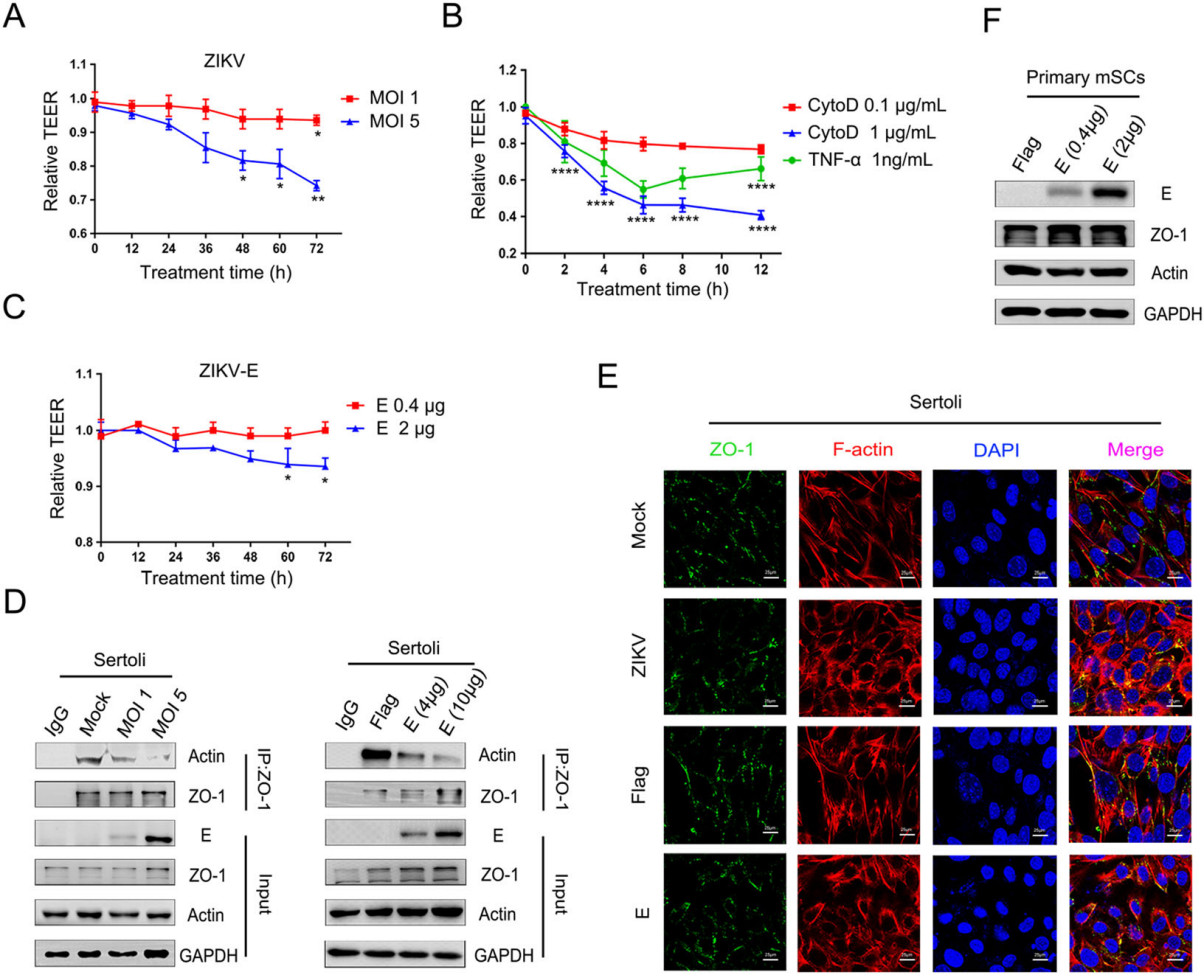
ZIKV E participates in the hyperpermeability of the *in vitro* mSCB model. **A** Effects of ZIKV infection on *in vitro* mSCB model. Primary mSCs were cultured on Transwell semipermeable membranes (0.4 μm pore size). Un-treated (mock) or treated with ZIKV at different MOIs when TEER values exceeds 50 Ω • cm^2^ and remains unchanged, detected TEER values at indicated time. Relative TEER: (Ωexperimental condition − Ωmedium alone)/(Ωmock − Ωmedium alone). The results are representative of three separate experiments. Each value represents the mean ± SD of 3 separate replicates. *, *P* < 0.05; **, *P* < 0.01 (two-way ANOVA). **B** Effects of the CytoD and TNF-α on *in vitro* mSCB model. CytoD (Control: DMEM containing 0.1% DMSO-treated) and TNF-α (Control: DMEM-treated) were treated on *in vitro* mSCB model and detected TEER values at indicated time. Relative TEER: (Ωexperimental condition − Ωmedium alone)/(Ωmock − Ωmedium alone). Each value represents the mean ± SD of 3 separate replicates. *P* values were analysed by comparing with the corresponding controls of 1 μg/mL CytoD and 1 ng/mL TNF-α respectively. ****, *P* < 0.0001 (two-way ANOVA). **C** Effects of ZIKV E overexprssion on *in vitro* mSCB model. Empty vector (mock) or pcDNA3.1(+)-Flag-E plasmid at different concentrations was transfected into mSCs, and TEER values were detected at different time points as indicated. Relative TEER: (Ωexperimental condition − Ωmedium alone)/(Ωmock − Ωmedium alone). The results are representative of three separate experiments. Each value represents the mean ± SD of 3 separate replicates. *, *P* < 0.05 (two-way ANOVA). **D** Co-IP of actin and ZO-1 after ZIKV infection and ZIKV E overexprssion. Sertoli cells were cultured overnight in 10 cm dishes, then un-treated (mock) or treated with ZIKV and transfected with empty vector or pcDNA3.1(+)-Flag-E plasmid at different concentrations, samples were collected after 48 h treatment. Cell lysate was immunoprecipitated with anti-ZO-1 or anti-mouse immunoglobulin G (IgG) antibody followed by SDS–PAGE and immunoblotting with anti-actin antibody. **E** Confocal microscopy analysis of the localization of ZO-1 after ZIKV infection and ZIKV E overexpression. Sertoli cells were un-treated (mock) or treated with ZIKV (MOI = 1) and transfected with 2 μg empty vector or 2 μg pcDNA3.1(+)-Flag-E plasmid. Then cells were fixed after 48 h treatment and stained with DAPI to label nuclei (blue), TRITC-phalloidin to label F-actin (red), and ZO-1 (green) were detected by anti-ZO-1 antibodies. The results are representative of three separate experiments. The scale bar indicates 25 μm. **F** Identification of E transfection efficiency in primary mSCs. Primary mSCs were transfected with empty vector or pcDNA3.1(+)-Flag-E plasmid and collected at 48 h after transfection, the expression of E, actin and ZO-1 was measured by WB.

According to previous studies, ZIKV has the ability to destroy the BTB, also called the SCB. Meanwhile, researches have implicated that TEER can be treated as a marker for the integrity of the BTB formed by the Sertoli cells (Qiu *et al.* 2016). Thus, we monitored changes in the TEER during viral infection of an *in vitro* mSCB model established using highly purified primary mSCs. ZIKV infection (MOI = 5) decreased the TEER slightly at 48 hpi and significantly at 72 hpi (Fig. 5A), which was consistent with our previous research (Hui *et al.* 2020). Moreover, we have previously confirmed that the decline in TEER was not the result of a decrease in cell viability caused by viral infection (Hui *et al.* 2020). Meanwhile, it has been reported that CytoD disrupts actin filaments and TJ barriers of intestinal epithelial cells (Fu *et al.* 2008), and that TNF-α mediated restructuring of the SCB *in vitro* involves actin cytoskeleton reorganization (Lydka *et al.* 2012). We observed a similar phenomenon in the *in vitro* mSCB model, CytoD or TNF-α treatment perturbed the SCB in a time- and dose-dependent manner when compared with the control treatment (Fig. 5B). In addition, a reduction in TEER was detected at 48 h post transfection in cells overexpressing a higher amount of protein E (Fig. 5C), indicating a direct effect of E protein on the BTB. These results demonstrated that both viral infection and ectopic expression of E protein could compromise the integrity of an mSCB model in a time- and dose-dependent manner, which may account for the testicular destruction observed in ZIKV-infected mice.

SCB function is supported by the binding of the organized actin cytoskeleton to adaptor proteins (Mok *et al.* 2013). As mentioned above, ZIKV infection and ectopically expressed E proteins induced the reorganization of F-actin network and decreased the TEER in an *in vitro* mSCB model. Thus, we hypothesized the disruption of the SCB was mediated by reorganization of F-actin. To validate this hypothesis, Co-IP and immunofluorescence assays were conducted. As shown in Fig. 5D, the association of actin with the TJ adaptor protein ZO-1 decreased in a dose-dependent manner in Sertoli cells after ZIKV infection or ZIKV E transfection, prompting that the weakened interaction between actin and ZO-1 might have contributed to the decrease in TEER. Furthermore, the continuous signal of ZO-1 at the cell–cell border was interrupted in infected or transfected Sertoli cells, as part of the proteins were translocated from the cell surface to the cytoplasm, when compared with control cells (Fig. 5E). As shown in Fig. 5F, overexpression of E in primary Sertoli cells did not affect the protein levels of actin and ZO-1. However, it is worth noticing that since F-actin was depolymerized and ZO-1 was diffused from the plasma membrane to the cytoplasm, the colocalization of the two proteins seems more obvious in treated cells. Nonetheless, these results suggested that the destruction of the organized actin cytoskeleton caused by ZIKV E contributed to the decline in TEER values, supporting the notion that ZIKV infection disrupts the SCB through the reorganization of F-actin, and identifying E protein as an important viral molecule in this process.

## Discussion

As noted, ZIKV infection leads to devastating diseases, such as congenital Zika syndrome. ZIKV RNA can be detected in semen collected several months after onset of symptoms of infection (Atkinson *et al.* 2017), and infectious virus was isolated in semen up to 69 days after symptom onset (Moreira *et al.* 2017). Thus, there is an urgent need to clarify the pathogenesis of ZIKV in the testes, especially, to identify the mechanisms underlying ZIKV breakage of the BTB.

Here, we sought to identify novel functions of ZIKV E and host interacting proteins in testicular Sertoli cells, which constitute the BTB and serve as a reservoir for ZIKV (Siemann *et al.* 2017). Based on LC–MS/MS data, we suspected an interaction between ZIKV EDIII and actin, which was validated by a Co-IP assay. We next generated E protein truncated constructs and confirmed that not only EDIII, but also sequences present in EDI and/or EDII interact with actin. This is consistent with findings in a previous study by Jitoboam *et al.* (2016), in which a construct containing only domains I and II of DENV2 E protein was capable of interacting with actin, suggesting that E protein harbors multiple actin interaction sites, and this may create a more favorable environment for ZIKV infection. Further, we found that the F-actin network was disorganized in the advanced ZIKV infection stage and after ectopic expression of E proteins in Sertoli cells, which is consistent with findings in DENV2-infected endothelial-like ECV304 cells (Wang *et al.* 2010). In addition, there have been reported that actin filaments are necessary for DENV infection (Zhang *et al.* 2016) and it can interact with DENV2 and DENV4 E proteins (Jitoboam *et al.* 2016). F-actin participates in the WNV maturation process (Chu *et al.* 2003), and is beneficial for JEV infection of IMR32 neuroblastoma cells (Henry Sum 2015). The interaction between ZIKV and actin found in this study suggests that flaviviruses commonly modulate the actin cytoskeleton.

Next, we treated Sertoli cells with actin filaments inhibitors to investigate whether or not the actin filament skeleton functions in ZIKV infection. A slight enhancement of virus invasion and extracellular virus particles production was observed in drugs-treated Sertoli cells, suggesting that CytoD or Jas acts to facilitate ZIKV penetration, which is the opposite of observations in other flaviviruses (Zhang *et al.* 2019). However, there has been controversy about the function of actin inhibitors, such as CytoB and CytoD, in viral infections. DENV2 invasion, assembly, and maturation were inhibited in CytoD-treated EAhy926 cells (Zhang *et al.* 2016) and WNV release was strongly (~ 10,000-fold) inhibited in CytoB-treated cells (Chu *et al.* 2003). Productive JEV infection was suppressed in CytoD-treated Vero cells (Nawa *et al.* 2003). The authors explained that the inhibition was due to disruption of the clathrin-mediated flavivirus invasion pathway in which actin filaments participate, and that an integral actin filaments skeleton is conducive to virus infection. However, there are also reports that CytoD treatment enhanced the infectivity of poliovirus and rotavirus (Deitch *et al.* 1973; Bass *et al.* 1995) and promoted the release of Newcastle disease virus from infected cells (Morrison and McGinnes 1985). In addition, CytoD has been reported to complement the disability of Nef-deleted HIV by promoting its infectivity (Campbell *et al.* 2004). Further, there are indications that CytoD enhances pseudorabies virus genome delivery to the nucleus (Jacob *et al.* 2015). It is worth noticing that the disruption of actin filaments by CytoD did not affect the entry process of WNV in mosquito cells (Chu *et al.* 2006).

Several reasons may account for these discrepancies: (1) Viruses and cells differed among studies. (2) CytoD or Jas is not a specific drug inhibiting virus entry, and it may trigger different signaling pathways to promote ZIKV invasion. (3) Although CytoD and Jas acted on actin network in different mechanisms, confocal immunofluorescence showed that both drugs depolymerized the existing normal actin filaments structure and destroyed the cell cortex barrier, which may facilitate ZIKV crossing the cortical actin barrier to promote its infection (Yoder *et al.* 2008; Delorme-Axford and Coyne 2011; Taylor *et al.* 2011). (4) Researches have showed that intact actin cytoskeleton structure is crucial for antiviral response during viral infection, it was reported that CytoD significantly inhibited the expression of IFN-β, IL-29, and TNF-α in influenza A virus-infected macrophages. This may also occur in drugs-treated Sertoli cells, the down-regulation of antiviral cytokines expression is conducive to ZIKV infection (Öhman *et al.* 2009). Moreover, although CytoD could inhibit HIV infection by destroying the actin skeleton (Iyengar *et al.* 1998; Yin *et al.* 2020), it could only exert an inhibitory effect when target cell receptor or co-receptor expression was low (Viard *et al.* 2002). We speculate that a similar phenomenon may have occurred in the Sertoli cells used in our study.

Interestingly, the treatment of CytoD and Jas reached a consistent result although they have different mechanisms on the actin network. We verified the results by blebbistatin, the myosin ATPase inhibitor, can also disrupt stress fibers without affecting other non-myosin involved actin structures. The virus invasion was slightly promoted with the increasing blebbistatin concentration though with no statistically significant difference (data not shown). It may be due to the fact that blebbistatin only acts on stress fibers, while CytoD or Jas treatment led to the global disruption of actin filaments dynamics and resulted in greater impacts than blebbistatin. This may be the reason for CytoD and Jas treatment are conducive to ZIKV infection with statistically significant.

Recently, there have several reports on the mechanisms of BTB disruption caused by ZIKV infection. Sheng *et al.* (2017) found that testicular TJ-associated proteins were down-regulated at the mRNA and protein levels after ZIKV infection. They surmised that the damage of TJs caused by ZIKV infection could compromise BTB function. However, Siemann *et al.* (2017) reported that ZIKV infection does not alter the expression of TJ and adherens junction proteins in human Sertoli cells. They inferred that Sertoli cells would be a reservoir for infection and that barrier integrity might not be directly affected after the virus infects Sertoli cells. Nevertheless, they showed that treatment of Sertoli cells with inflammatory mediators produced by ZIKV-infected macrophages led to degradation of ZO-1 protein, which may contribute to increased SCB permeability. Additionally, up-regulation of cell adhesion molecules benefited peripheral immune cells in crossing the barrier, further aggravating the inflammatory response and eventually destroying the BTB (Siemann *et al.* 2017). We recently reported the molecular mechanisms underlying testis invasion by ZIKV (Hui *et al.* 2020). Our previous study revealed that matrix metalloproteinase 9 (MMP9) was up-regulated by ZIKV infection in cultured primary mSCs and A129 mice. The MMP9 protein level was elevated by nonstructural protein 1 (NS1), and the interaction between MMP9 and NS1 induced K63-linked polyubiquitination of MMP9. The accumulation of MMP9 led to the degradation of TJ proteins and type IV collagen, which are essential in BTB maintenance, thus leading to the hyperpermeability of BTB (Hui *et al.* 2020).

Previous studies have revealed that different molecules, such as Rictor/mTORC2 or ribosomal protein S6, a major regulator of F-actin organization and adhesion protein recruitment at the BTB, regulate the tightness of BTB through effects on F-actin (Mok *et al.* 2012, 2013). The environmental toxins CdCl_2_ and bisphenol A disrupted the localization of adhesion proteins between human Sertoli cells by restructuring F-actin, thereby disrupting the BTB, interfering with normal sperm development, and reducing the number and quality of sperm, leading to male infertility (Xiao *et al.* 2014). Moreover, disruption of the actin cytoskeleton compromises the blood–brain barrier (Al-Obaidi *et al.* 2018). Accordingly, we observed restructuring of the actin cytoskeleton after ZIKV infection or E protein overexpression in Sertoli cells, suggesting that actin cytoskeleton disruption is involved in the interruption of the BTB by ZIKV infection or overexpression of the E protein. In support of this, TEER analysis revealed hyperpermeability of an *in vitro* mSCB model after ZIKV infection and ZIKV E overexpression, Co-IP showed that a weakened correlation between ZO-1 and actin. Besides, in combination with the results of confocal microscopy in Fig. 3 and Fig. 5E, we found that actin was not organized in actin filaments, but dispersed in the cytoplasm, or part of it may be recruited to the plasma membrane after ZIKV infection or E ectopic expression. These changes revealed that actin no longer provides strong support to BTB in the form of actin filaments (Mok *et al.* 2013), resulted in trans-localization of ZO-1 from the plasma membrane to the cytoplasm, which may disturb interactions between ZO-1 and TJ or adherent junction proteins. These phenomena may, at least in part, explain the compromised BTB integrity, and suggest a possible correlation between the accumulation of ZIKV E protein and BTB dysfunction.

While we did not prove a direct interaction between E protein and actin, previous report has identified the association of ZO-1 and actin (Mok *et al.* 2013). Whether the interaction between E protein and actin is mediated by ZO-1 or other proteins requires further investigation. In addition, actin is a skeleton portion in cytosol while E protein is the envelope protein of ZIKV and supposed to present in compartments along the secretion pathway, although there have been many studies on the correlation between flaviviruses and the host cytoskeleton, especially, the interaction between dengue virus E protein and actin filaments have been proved (Wang *et al.* 2010; Jitoboam *et al.* 2016), the possible mechanisms under which these two proteins could access to each other has not yet been elucidated. Based on the previous findings, we surmised the possible mechanism as below: it is known that actin filaments constitute part of the cytoskeleton in three-dimensional and participate in protein synthesis and cargo transport (Muñoz-Lasso *et al.* 2020). Meanwhile, studies have showed that ZIKV E protein protrudes on the virus surface and undergoes positional reorganization during the virus life cycle (Shi and Gao 2017; Shi *et al*. 2018), these may increase the chance of contact and the interaction of the two.

Collectively, our findings provided new mechanisms by which ZIKV crosses the BTB and possibly, other blood-tissue barriers, and pave the way for the development of preventative strategies against ZIKV testicular infection and treatments for testis damage caused by this devastating infection.
